# TRPA1 channel mediates organophosphate-induced delayed neuropathy

**DOI:** 10.1038/celldisc.2017.24

**Published:** 2017-08-01

**Authors:** Qiang Ding, Sui Fang, Xueqin Chen, Youxin Wang, Jian Li, Fuyun Tian, Xiang Xu, Bernard Attali, Xin Xie, Zhaobing Gao

**Affiliations:** 1 CAS Key Laboratory of Receptor Research, State Key Laboratory of Drug Research, Shanghai Institute of Materia Medica, Chinese Academy of Sciences, Shanghai, China; 2 University of Chinese Academy of Sciences, Beijing, China; 3 Shanghai Leado Pharmatech Co. Ltd, Shanghai, China; 4 Shanghai Key Laboratory of New Drug Design, School of Pharmacy, East China University of Science and Technology, Shanghai, China; 5 Department of Physiology & Pharmacology, Sackler Faculty of Medicine, Tel Aviv University, Tel Aviv, Israel

**Keywords:** organophosphate, malathion, TOCP, OPIDN, TRPA1

## Abstract

The organophosphate-induced delayed neuropathy (OPIDN), often leads to paresthesias, ataxia and paralysis, occurs in the late-stage of acute poisoning or after repeated exposures to organophosphate (OP) insecticides or nerve agents, and may contribute to the Gulf War Syndrome. The acute phase of OP poisoning is often attributed to acetylcholinesterase inhibition. However, the underlying mechanism for the delayed neuropathy remains unknown and no treatment is available. Here we demonstrate that TRPA1 channel (Transient receptor potential cation channel, member A1) mediates OPIDN. A variety of OPs, exemplified by malathion, activates TRPA1 but not other neuronal TRP channels. Malathion increases the intracellular calcium levels and upregulates the excitability of mouse dorsal root ganglion neurons *in vitro*. Mice with repeated exposures to malathion also develop local tissue nerve injuries and pain-related behaviors, which resembles OPIDN. Both the neuropathological changes and the nocifensive behaviors can be attenuated by treatment of TRPA1 antagonist HC030031 or abolished by knockout of *Trpa1* gene. In the classic hens OPIDN model, malathion causes nerve injuries and ataxia to a similar level as the positive inducer tri-ortho-cresyl phosphate (TOCP), which also activates TRPA1 channel. Treatment with HC030031 reduces the damages caused by malathion or tri-ortho-cresyl phosphate. Duloxetine and Ketotifen, two commercially available drugs exhibiting TRPA1 inhibitory activity, show neuroprotective effects against OPIDN and might be used in emergency situations. The current study suggests TRPA1 is the major mediator of OPIDN and targeting TRPA1 is an effective way for the treatment of OPIDN.

## Introduction

Organophosphates (OPs) are the active ingredient of many insecticides, herbicides, and nerve agents and are also widely used as solvents, plasticizers, and additives in industry. Approximately three million individuals are exposed to OPs worldwide per year [1]. Organophosphate-induced delayed neuropathy (OPIDN), which often leads to paresthesias, ataxia and paralysis, occurs during the late-stage of acute poisoning or after repeated exposure to OPs [2, 3]. The OP-induced neuropathy may also contribute to the Gulf War Syndrome [4, 5]. The acute phase of OP poisoning is usually attributed to acetylcholinesterase (AchE) inhibition [6, 7]. However, the mechanism underlying the neuropathy remains elusive, and no treatment is available. The patients suffering OPIDN and the increasing risk of exposure to OP-type nerve agents, such as sarin and tabun, during military conflicts or terrorist attacks, make it urgent to develop effective preventions and therapeutics for this OP-induced neuropathy [8].

OPs could be divided into two major groups according to their inhibitory activity on AchE. Most OPs, including insecticides, herbicides and nerve agents, show potent inhibition of AchE. It has been demonstrated that these cholinergic OPs can induce OPIDN [9, 10]. In contrast, tri-ortho-cresyl phosphate (TOCP) is an OP that lacks AchE inhibitory activity and is widely used as an additive in industry. Consumption of Jamaica ginger extract adulterated with TOCP led to 'ginger jake paralysis' in an estimated 50,000 Americans in 1930s [11]. Later, similar paralysis-causing OPIDN occurred in Morocco, India and France due to the consumption of cooking oil contaminated with mineral oil containing TOCP [12,13,14,15,16]. The latest large-scale outbreak of OPIDN that resulted from the consumption of flour contaminated by TOCP occurred in China in 1990 [17]. The fact that TOCP could also induce OPIDN further suggests that the delayed neuropathy is caused by mechanisms other than AchE inhibition.

In previous studies, the neuropathy target esterase (NTE) has been proposed to be a potential mediator for OPIDN [18,19,20]. NTE is a large esterase of 1,327 amino acids that is different from AchE. The inhibition and subsequent aging (dealkylation) of NTE were observed in the early stage of OPIDN [18,19,20]. However, NTE inhibition cannot explain all aspects of OPIDN [9, 18,19,20,21]. Some NTE inhibitors, such as ethyl carbamate, butylate and benzenesulfonyl chloride, were further found to aggravate the OP-induced neuropathy [22, 23]. In the current study, we demonstrate that TRPA1 (Transient receptor potential cation channel, member A1) is a major mediator of OPIDN. Being a member of the superfamily of TRP ion channels, TRPA1 is originally identified as a polymodal sensor and can be activated by plenty of noxious stimuli, including thermal (cold) nociception, mechanical stimulus and environmental irritants [24,25,26,27]. As a Ca^2+^ permeable non-selective cation channel, TRPA1 has been demonstrated to function in a variety of physiological or pathological processes, such as cold sensation [26], coughing [28], oxygen sensation [29], itching [30, 31] and inflammatory pain sensation [32]. TRPA1 is thought to be an exciting target for the treatment of various pain states as well as allergic asthma [32]. In a chemical screening to identify new modulators of TRPA1 channels, we unexpectedly discovered that a number of OPs could activate TRPA1 channels. Further studies demonstrated that TRPA1 is a major mediator of OPIDN. Pharmacological inhibition of TRPA1 or knockout of *Trpa1* gene significantly alleviates the OP-induced neuropathy.

## Results

### Malathion is a selective TRPA1 activator

**Figure 1:**
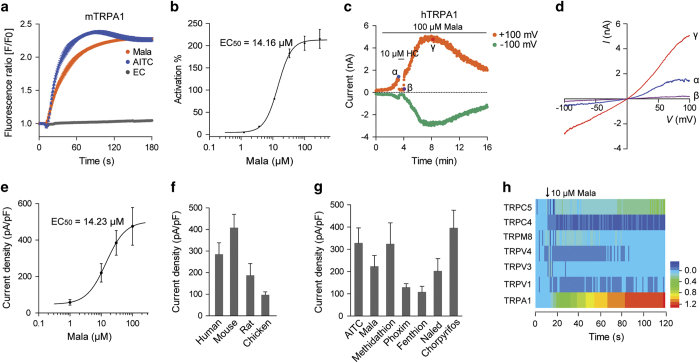
Malathion activates TRPA1 channels. (**a**) Time course of fluorescence signals induced by 10 μM malathion (Mala) or 10 μM AITC in HEK293 cells expressing mTRPA1. The fluorescence signals are scaled as F/F_0_ (*n*=5). EC indicates extracellular solution. (**b**) Dose–response curve showing malathion-induced activation of mTRPA1 (*n*=8–10). (**c**) Whole-cell currents of a HEK293 cell expressing hTRPA1 in calcium-free extracellular solution. HC030031 (HC, 10 μM) was applied to validate the hTRPA1-mediated currents. The currents measured at −100 mV (green circles) and +100 mV (orange circles) during each ramp were plotted as a function of time. (**d**) The current-voltage (*I*–*V*) relationships of malathion-induced currents. The blue, purple and red lines were measured at the time points α, β and γ (shown in **c**), respectively. (**e**) Dose–response curve showing the malathion-induced currents of hTRPA1 (*n*=8–10). (**f**) The current densities of human, mouse, rat and chicken TRPA1 channels induced by 30 μM malathion (*n*=5). (**g**) The current density of hTRPA1 induced by AITC or the indicated OPs (30 μM, *n*=5–7). (**h**) Heat map showing the time course of the 10 μM malathion-induced fluorescence signals of TRPA1, TRPV1, TRPV3, TRPV4, TRPM8, TRPC4 and TRPC5 channels. The statistical data are presented as the mean±s.e.m.

Malathion is a commonly used OP insecticide and one of major poisons leading to OP intoxication. A number of malathion-induced human OPIDN have been reported [33, 34]. Similar to allyl isothiocyanate (AITC), a classic TRPA1 agonist, malathion was found to dose-dependently activate mTRPA1 channels expressed in HEK293 cells with an EC_50_ of 14.16±1.02 μM, as determined by measuring the change in [Ca^2+^]_i_ (Figure 1a and b). Several other OPs that often lead to human intoxication, including methidathion, phoxim, fenthion, naled and chlorpyrifos, were also found to activate mTRPA1 channels, with EC_50_ values ranging from 10 μM to 40 μM (Table 1). The agonistic effect of malathion was further examined in TRPA1 channels cloned from various species using whole-cell patch-clamp recording. Mock-transfected cells did not respond to the OP, whereas cells expressing TRPA1 showed a large activation current, regardless of whether the channels were derived from mouse (m), rat (r), chicken (c) or human (h) (Figure 1c–f; Supplementary Figure S1). The malathion-elicited currents exhibited typical properties of TRPA1 currents: they could be inhibited by HC030031 and had a reversal potential of 0 mV (Figure 1d; Supplementary Figure S1). The EC_50_ of malathion on hTRPA1 was 14.23±1.84 μM, when measured by whole-cell patch-clamp (Figure 1e), very close to the value measured by calcium assay. Other tested OPs elicited similar whole-cell currents in HEK293 cells expressing hTRPA1 as malathion did (Figure 1g; Supplementary Figure S2). In addition to TRPA1, a number of other neuronal TRP channels, such as TRPV1, TRPV3, TRPV4, TRPM8, TRPC4 and TRPC5, are also expressed in peripheral sensory neurons [35, 36]. Malathion could not activate these TRP channels, except TRPA1. In contrast, these TRP channels could be activated by their respective agonists (Figure 1h; Supplementary Figure S3). These data indicate that malathion specifically activates TRPA1 channels.

### Molecular determinants of malathion activating TRPA1 channels

**Figure 2:**
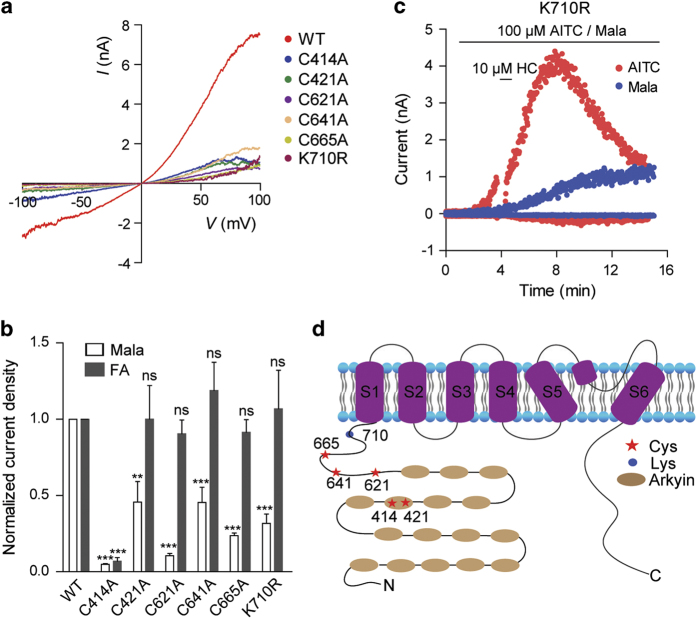
The mechanism of malathion-mediated TRPA1 activation. (**a**) The *I*–*V* plots of WT and mutant hTRPA1 currents activated by 30 μM Mala. (**b**) Comparison of the responses of WT and mutant hTRPA1 channels with 30 μM malathion and 30 μM flufenamic acid (FA) (*n*=5–9). (**c**) Whole-cell currents of the mutant K710R in the presence of 100 μM AITC and 100 μM malathion. HC030031 (HC, 10 μM) was applied to validate the hTRPA1-mediated currents. (**d**) Schematic representation of the structure of hTRPA1 and the essential residues for malathion activity. The statistical data are presented as the mean±s.e.m. *P<0.05, **P<0.01 and ***P<0.001.

We next conducted mutagenesis studies to explore the molecular basis for the malathion-induced activation of TRPA1 channel. AITC activates TRPA1 through reactivity-based covalent modification [37, 38]. Previous studies have revealed multiple critical residues, including five cysteines (C414, C421, C621, C641 and C665) and a lysine (K710), for AITC-induced hTRPA1 activation. Similar to AITC, malathion and other OPs also contain an electrophilic center: a phosphorus atom (Table 1). We hypothesized that the residues important for AITC activity may also play a role in malathion-mediated hTRPA1 activation. Indeed, although hTRPA1 carrying single mutations of these residues (C414A, C421A, C621A, C641A, C665A or K710R) were functional, their responses to malathion were significantly reduced compared to those of WT channels (Figure 2a and b; Supplementary Figure S4). In contrast, these residues (except C414) were not essential for the activation of TRPA1 channels by flufenamic acid, a non-electrophilic TRPA1 agonist [39] (Figure 2b). These data suggest that the molecular determinants that are critical for AITC-mediated TRPA1 activation are also important for malathion’s effects on TRPA1. Interestingly, although the K710R mutation reduced the maximal current and prevented the inactivation of malathion-activated TRPA1 channels, it did not influence the inactivation of the AITC-activated channels; these results reveal a distinct feature of malathion-mediated activation of TRPA1 channels (Figure 2c and d).

### Malathion induces calcium influx and enhances neuronal excitability in native neurons

**Figure 3:**
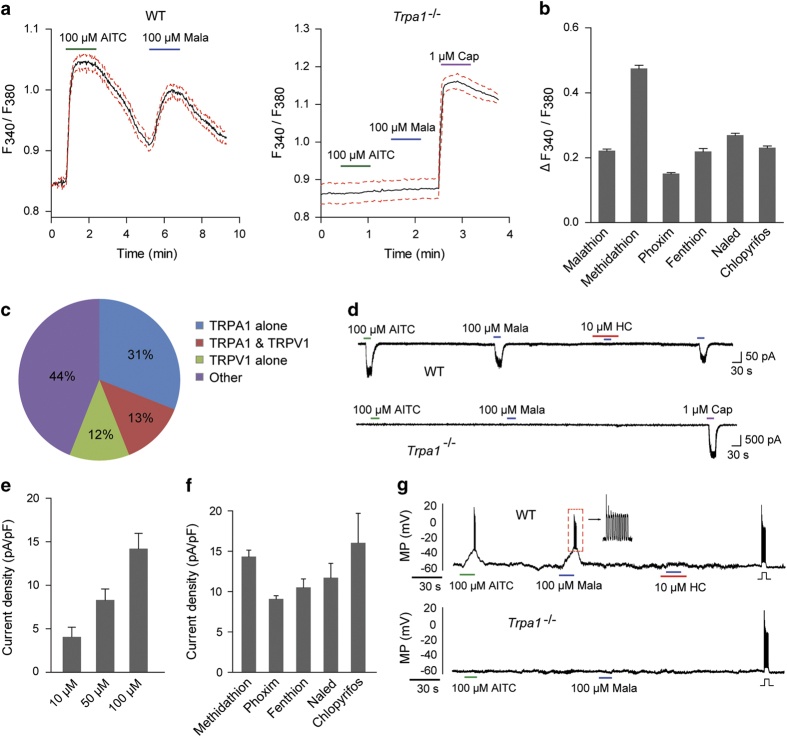
Malathion elicits inward currents and induces action potential firing in small-diameter DRG neurons via TRPA1 channels. (**a**) Changes in the fluorescence ratio in DRG neurons from WT (left) and *Trpa1*^*−/−*^ mice (right) after sequential application of AITC and Mala. Capsaicin (Cap) was used to verify the functionality of the *Trpa1*^*−/−*^ DRG. The green, blue and purple bars indicate the AITC, malathion and capsaicin application periods, respectively. (**b**) Summary of fluorescence ratio changes elicited by 100 μM of the indicated OPs in WT DRG neurons (*n*=5–7). (**c**) Percentages of TRPA1- or TRPV1-positive DRG neurons from WT mice. (**d**) Inward currents induced by 100 μM AITC or malathion in DRG neurons. Note that malathion-induced inward currents were blocked by 10 μM HC030031 (HC) and abolished in *Trpa1*^*−/−*^ mice (*n*=7). (**e**) Dose-dependent effects of malathion on DRG neurons from WT mice (*n*=3–4). (**f**) Summary of current density induced by the indicated OPs (100 μM) in WT DRG neurons (*n*=3–4). (**g**) Action potential firing induced by 100 μM AITC or malathion in DRG neurons (*n*=4). Note that malathion-induced action potential firings were blocked by 10 μM HC030031 and eliminated in *Trpa1*^−/−^ mice. The data represent the mean±s.e.m.

TRPA1 is expressed in a subpopulation of primary sensory neurons of the trigeminal, nodose, and dorsal root ganglia (DRG) [40]. In DRG, according to a newly-developed cataloging method, expression of TRPA1 was detected in five clusters or subclusters among a total of 14 neuronal subclusters [41]. These TPRA1-positive neurons participate in pain, thermal and mechanoreceptive sensation [41]. Isolated mouse DRG neurons were stimulated with malathion and changes in [Ca^2+^]_i_ were monitored. Among the tested wild-type small-diameter neurons, ~44% (197/451) neurons displayed a robust increase in [Ca^2+^]_i_ after sequential application of AITC and malathion (Figure 3a–c). In contrast, the DRG neurons from *Trpa1*^−/−^ mice were thoroughly unresponsive to malathion and AITC, although some of these neurons retained responses to capsaicin, a selective agonist of TRPV1 channels (Figure 3a, right panel). We then tested whether malathion could elicit currents in isolated DRG neurons. Consistent with the [Ca^2+^]_i_ measurements, robust inward currents could be induced by malathion in a dose-dependent manner in ~39% (7/18) of the tested small-diameter neurons from the WT mice (Figure 3d and e). These induced currents could be inhibited by HC030031 (Figure 3d, top panel). In contrast, both malathion and AITC failed to induce currents in neurons (17/17) from *Trpa1*^*−/−*^ mice, although some of the tested neurons exhibited sensitivity to capsaicin (Figure 3d, bottom panel). Whether the excitability of DRG neurons could be affected by malathion was further investigated. In WT small-diameter neurons, applying 100 μM AITC or malathion-elicited bursts of action potentials, which were suppressed by HC030031 and abolished in *Trpa1*^*−/−*^ neurons (Figure 3g). At the end of each recording, a 20 pA inward current was injected to validate the viability of the recorded neurons. Other tested OPs elicited increases in [Ca^2+^]_i_ and inward currents similar to those induced by malathion (Figure 3b and f; Supplementary Figures S5 and S6). These data demonstrate that, in primary sensory neurons, malathion induces calcium influx and enhances excitability, two proven causes of neuronal damage [42, 43], by activating TRPA1 channels.

### TRPA1 deficiency or blockage alleviates malathion-induced pain and nerve injury in mice

**Figure 4:**
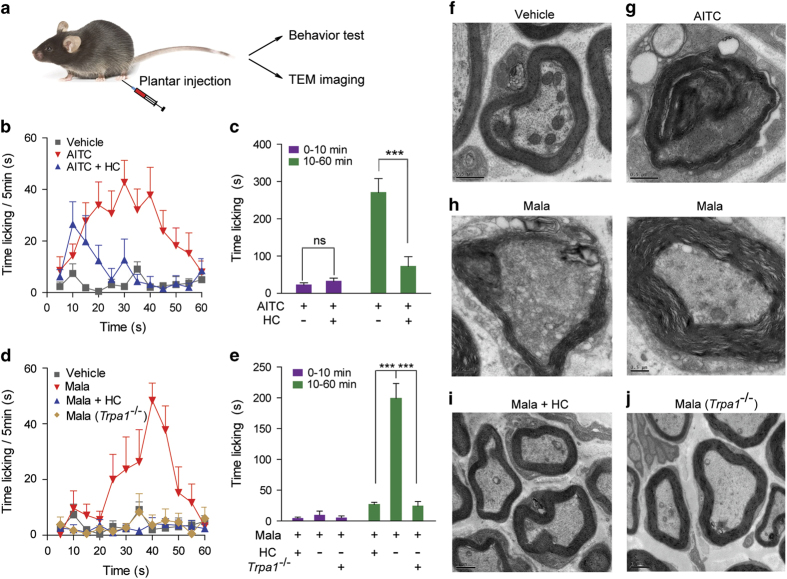
TRPA1-KO or blockage alleviates malathion-induced nociception and nerve injury in mice. (**a**) Schematic diagram of the mouse tests. AITC (10 μl, 5%) or malathion (10 μl, 30%) were given by intraplantar route, followed by behavioral test and TEM imaging. (**b**) Time course of licking in mice treated with 5% AITC (red triangles, *n*=10). Mice that were pretreated with HC030031 (HC, 200 mg kg^−1^, per oral, blue triangles, *n*=9) licked for significantly shorter periods than AITC-treated mice. (**c**) Quantification of the AITC response binned into different phases. Phase I (0–10 min); Phase II (10–60 min). (**d**) As in (**b**) but also including a malathion (Mala)-treated *Trpa1*^*−/−*^ group, *n*=8–10 per group. (**e**) Quantification of the malathion response during the different phases for the experimental groups. (**f**–**j**) Ultrastructural image of local nerve fibers from WT mice in the vehicle (**f**), 5% AITC (**g**), 30% malathion (**h**), malathion pretreated with HC030031 (**i**) and *Trpa1*^−/−^ mice treated with 30% malathion (**j**). For the panel **h**, two types of malathion-induced neuropathies were displayed, that is, loss of the myelin sheath (left) and avulsion (right). Scale bar is 0.5 μm for the vehicle, AITC and malathion groups and 1 μm for the malathion+HC and malathion (*Trpa1*^−/−^) groups. The data represent the mean±s.e.m. ***P<0.001.

Activation of TRPA1 channels expressed in peripheral nociceptors triggers pain in rodents [32]. By measuring paw-licking time in mice, plantar injection of AITC was found to elicit nociception-like response in the early stage (<10 min) and the response gradually increased and became maximal at 30 min (Figure 4a and b). This AITC-evoked nocifensive behavior could be suppressed by treatment of HC030031 (Figure 4b and c). Interestingly, plantar injection of malathion evoked a nociception-like behavior similar to that evoked by AITC, which could also be blocked by HC030031 treatment (Figure 4d and e). More importantly, the malathion-induced nociception-like behavior was not observed in *Trpa1*^*−/−*^ mice (Figure 4d and e). Peripheral nerve fibers, including both myelinated and unmyelinated fibers, are responsible for the nociceptive signaling transduction [41]. Peripheral nerve fiber damage is a typical feature of OP-induced neuropathy [9, 10]. Potential malathion-induced nerve fiber damage *in vivo* was observed using transmission electron microscopy. Plantar injection of malathion or AITC induced similar damage of the myelin sheath of nerves in the tissue surrounding the injection site, including a certain degree of avulsion and partial or total loss of the myelin sheath (Figure 4g and h). In contrast, the myelin sheaths of nerve fibers were intact in the vehicle group (Figure 4f). HC030031 pretreatment or *Trpa1* knockout significantly protected the myelin sheath (Figure 4i and j). These results support that TRPA1 channels expressed in primary nociceptors play an essential role in malathion-induced pain and neuropathy.

### TOCP activates TRPA1 channels

**Figure 5:**
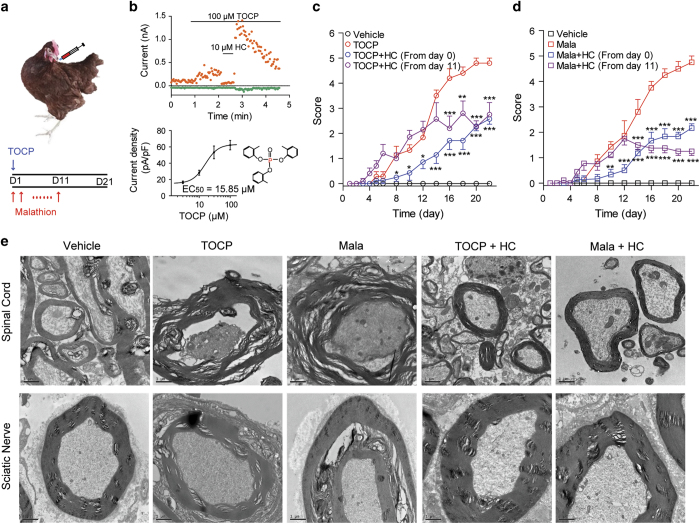
Pharmacological inhibition of TRPA1 alleviates OPIDN in hens. (**a**) Schematic representation of the administration of TOCP and malathion to hens. (**b**) Whole-cell currents of a HEK293 cell expressing cTRPA1 channels elicited by 100 μM TOCP in calcium-free external solution (upper) and the dose–response curve of the TOCP activation effect (lower) along with the structure of TOCP (*n*=4–8). (**c**) Time course of the changes of OPIDN scores following vehicle (black, *n*=6), TOCP (750 mg kg^−1^, red, *n*=8), TOCP+HC030031 (100 mg kg^−1^) from day 0 (blue, *n*=8) and from day 11 (purple, *n*=8). (**d**) Time course of the changes of the neuropathy scores of hens following vehicle (black, *n*=6), malathion (75 mg kg^−1^, red, *n*=8), malathion+HC030031 (100 mg kg^−1^) from day 0 (blue, *n*=8) and from day 11 (purple, *n*=8). The clinical symptoms of the delayed neuropathy was estimated in hens daily for 21 days using a five-point scale. (**e**) TEM image of hen spinal cords (upper row) and sciatic nerves (lower row) from the various groups (10 000×). For panels (**c**) and (**d**), two-way ANOVA was used for statistical analysis, *P<0.05, **P<0.01, ***P<0.001.

Hen is the classical model animal to study OPIDN [16, 44]. Typically, hens’ model of OPIDN is induced by TOCP (Figure 5a), which also causes OPIDN in human [16, 44]. So we tested whether TOCP could activate TRPA1. As expected, whole-cell patch-clamp showed that TOCP could also effectively activate human, mouse, rat or chicken TRPA1 channels (Figure 5b; Supplementary Figure S7a–d). The EC_50_ of TOCP on cTRPA1 was 15.85±1.37 μM (Figure 5b), which was comparable to the EC_50_ of malathion (10.75±1.68 μM) (Supplementary Figure S7e).

### Pharmacological inhibition of TRPA1 channels suppresses OPIDN

In hens, a single dose (750 mg kg^−1^) of TOCP can induce typical delayed neuropathy, including both neuropathological injuries and neurological abnormalities, similar to those found in human OPIDN [16, 44]. Malathion can potently inhibit AchE, and therefore, to avoid death induced by acute toxicity, a low dose of malathion (75 mg kg^−1^, q.d.) was given to adult hens for 11 days (Figure 5a). On day 5, slow and clumsy gait was observed in both the malathion and TOCP groups (Figure 5c and d), and the clinical symptoms of both groups worsened over time. All hens in the TOCP-treated group were completely paralyzed by the end of the 21-day experimental period. In contrast, pharmacological inhibition of TRPA1 channels had significant neuroprotective effects. HC030031 (100 mg kg^−1^) was given daily by gavage at the beginning of each experiment (day 0) or after the onset of the neuropathy (day 11). Clinical signs of the delayed neuropathy induced by either TOCP or malathion, particularly the features of ataxia, were significantly alleviated in the HC030031-treated groups (Figure 5c and d). Notably, HC030031 effectively suppressed the increasing trend of the clinical scores, even if it was administrated after onset of the disease. In addition to peripheral nerve fiber damages, the central nerve system damages have also been observed in both human and hens OPIDN [45, 46]. The neuropathological changes in spinal cord and sciatic nerve were consistent with the clinical scores (Figure 5e; Supplementary Figure S8). Both malathion and TOCP severely damaged the spinal cord and sciatic nerve, inducing large-scale avulsion and dissolution of the myelin sheaths, causing atrophy or content loss of the nerve fibers and leading to the accumulation of double-membrane autophagosomes in axons. Owing to the abnormal accumulation of autophagic vacuoles and autophagosomes, the damaged axons displayed swellings. All of these nerve injuries were significantly reduced in HC030031-treated groups. These results indicate that pharmacological inhibition of TRPA1 channels is an effective means to suppress OPIDN.

### Duloxetine and ketotifen suppress OPIDN

**Figure 6:**
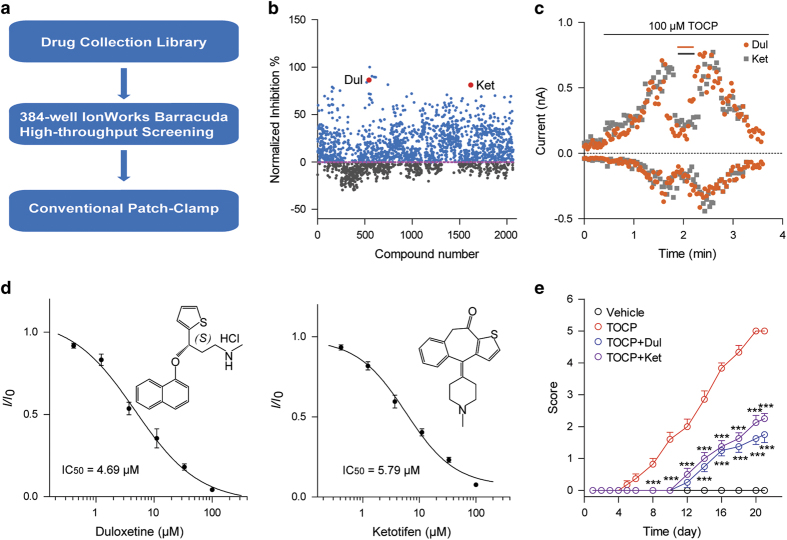
Duloxetine and ketotifen screened by high-throughput system alleviate OPIDN in hens. (**a**) The route diagram of TRPA1 inhibitor detection. (**b**) Scatter statistics of TRPA1 channel inhibitory activity of the drug collection library. Duloxetine (Dul) and ketotifen (Ket) are displayed in red dot. (**c**) Representative currents showing the inhibitory effects of 30 μM Dul or 30 μM Ket on the TOCP-induced cTRPA1 currents. (**d**) Dose–response curves of Dul (left) and Ket (right) on cTRPA1 channels (*n*=4–8). (**e**) Time course of OPIDN scores in hens following the indicated treatments. TOCP was administered in the same manner as described in the panel Figure 5a. Dul (100 mg kg^−1^) and Ket (100 mg kg^−1^) were administered daily by gavage from day 0. *n*=8 for each group. For panel (**e**), two-way ANOVA was used for statistical analysis, ***P<0.001.

A number of drug candidates targeting TRPA1 channels are currently in clinical trials [47, 48]. However, to date, none of them has been launched. To meet the urgent needs for TRPA1 antagonists in clinical practice, we screened an FDA-approved drug library using a high-throughput automated patch-clamp IWB (Figure 6a). The IWB is a 384-well planar-array platform that can simultaneously record 384 currents. Instead of monitoring the [Ca^2+^]_i_ changes in the fluorescence-based calcium assay, the newly-developed method could directly measure the TRPA1 currents before and after administration of compounds, which makes the screening more efficient and minimizes false positive hits. We screened ~2 000 commercially available drugs and identified a number of new antagonists of TRPA1 channels (Figure 6b). These hits were further validated using manual whole-cell patch-clamp. Among them, duloxetine, an anti-depressive drug, and ketotifen, an anti-histamine, were demonstrated to inhibit TRPA1 channels at micromolar level (Figure 6c and d). The IC_50_ of the duloxetine and ketotifen on cTRPA1 channels were 4.69±1.40 μM and 5.79±1.18 μM, respectively. The protective activities of these two drugs in OPIDN were then evaluated in hens. Similar with HC030031, both drugs significantly alleviated TOCP-induced symptoms in hens (Figure 6e). Duloxetine and ketotifen could meet the urgent needs of clinical practice.

## Discussion

OPs are the most commonly used insecticides in the world [49]. OP-type insecticides can be absorbed by almost all routes, including inhalation, ingestion and dermal absorption. In addition, OP-type insecticides are also frequently used to commit suicide [1, 50]. The main mechanism of OPs poisoning is the inhibition of AchE. Once AchE is inactivated in human, the accumulated acetylcholine throughout the nervous system will cause acute cholinergic syndrome and may lead to death [51]. Worldwide mortality rate of OP acute poisoning is ~3–25% [52]. Since the onset of the symptoms is often within minutes to hours, the patients require immediate emergency room treatment to prevent the fatal outcome. For the treatment of the acute poisoning, the standard antidotes include a pralidoxime to reactivate AchE and an anticholinergic such as atropine [53, 54]. It is interesting to note, although these antidotal therapies yield a remarkable survival rate, they fail to prevent OPIDN. OPIDN usually occurs 1–5 weeks after the absorption of large doses of OPs, regardless of whether these OPs have AchE inhibitory activity or not [17, 55]. Repeated or prolonged exposure of OPs, even at relatively low levels and without causing acute symptoms, may also result in the neuropathy [56]. Ataxia and paralysis are the typical symptoms of OPIDN [57]. Other reported symptoms include lower limb numbness, weakness, pain, meroparesthesia and amyotrophia [58, 59]. Although peripheral axonal degeneration and demyelination are one typical feature of OPIDN, damage to the central nervous system may also occur [60]. Different from the acute toxicity that mainly stems from the inhibition of AchE, the mechanism underlying the delayed neuropathy remains unclear and no treatment is available.

In the present study, we provide evidence that TRPA1 is a major mediator of OPIDN. All the tested OPs, exemplified by malathion, potently activate TRPA1 channels in both recombinant cells and native DRG neurons. In mice, administration of either malathion or AITC, a classic agonist of TRPA1, causes OPIDN-like neuropathological injuries. Knockout of gene or pharmacological inhibition of TRPA1 channels significantly alleviates these nerve injuries and behavioral changes. Due to that the sensitivity and intoxication symptoms are similar to those of human beings, hen is widely used to study OPIDN [44, 61, 62]. TOCP, the classic inducer of OPIDN but lacks inhibitory activity of AchE, exhibits significant agonistic activity on TRPA1 channels. As reported in previous studies, NTE could be a partial contributor to OPIDN [9, 21]. Notably, we found that those NTE inhibitors also exerted significant agonistic activity on TRPA1 channels (Supplementary Figure S9), which could explain how they aggravate the OP-induced neuropathy and is consistent with the major role of TRPA1 in OPIDN.

TRPA1 is a channel permeable to Ca^2+^. Potential roles of Ca^2+^ in OPIDN has also been noticed in earlier studies. Initially, decrease of free Ca^2+^ concentration in serum was observed [63]. The imbalance of intracellular and extracellular Ca^2+^ concentration was thus speculated to be a potential factor in the neuropathy. However, Ca^2+^ supplementation failed to prevent the TOCP-induced neuropathy, although the Ca^2+^ concentration in serum has been elevated in some supplementation groups [63]. Paradoxically, some voltage-gated Ca^2+^ channel blockers, exemplified by verapamil, were beneficial when they were used alone or co-administrated with Ca^2+^ supplementation together [44, 64, 65]. Intriguingly, we found that verapamil is also an inhibitor of TRPA1 channels (Supplementary Figure S10). Verapamil thus might perform neuroprotective effects by inhibiting TRPA1-mediated Ca^2+^ influx as HC030031 does. The OP-induced elevation of [Ca^2+^]_i_ in native neurons is in line with the essential roles of TRPA1-mediated Ca^2+^ influx for myelin damage in ischemia [66]. Blocking of the TRPA1- but not other Ca^2+^ permeable channels-mediated Ca^2+^ entry largely reduces ischemic damage to myelin [66]. Therefore, our study provides new insight for the effects of TRPA1-mediated Ca^2+^ influx in neuropathological conditions.

TRPA1 channels could be activated by many exogenous chemical molecules, including AITC and OPs. Being the active ingredient of mustard plant family, AITC is a classic selective agonist of TRPA1. In the current study, we showed that OPs selectively activate TRPA1 for the first time. Interestingly, human beings or rodents do not present the neuropathy symptoms even after intake of a large amount of AITC. We suspect the possible reasons might include: (a) AITC is usually ingested through the digestive tract as a food composition, which will be hydrolyzed by the acidic water environment in the stomach [67, 68]. Under this condition, AITC cannot reach the effective concentration to induce damage in the nervous tissue. As one supportive evidence, local injection of AITC indeed generated similar neuropathy as OPs did (Figure 4g and h). (b) The patients with OPs poisoning often absorb extremely high dose of toxicant [69, 70], and quite a part of these toxicants could be stored in fat tissues and may cause lasting TRPA1 activation. In addition, whether the long-term exposure to OPs would influence the expression of TPRA1 *in vivo* is also worth further investigation.

Pharmacological inhibition of TRPA1 channels is an effective strategy for OPIDN prevention and treatment. However, to date, there is no drug specifically targeting TRPA1 in the market. HC030031, a selective TRPA1 antagonist, shows efficacy in inflammatory pain models but has been terminated in preclinical trial. Multiple drug candidates targeting TRPA1 are under active development. HX-100 is one of TRPA1 antagonists that are currently in Phase I study [47]. In the preclinical trials, HX-100 exhibits promising efficacy in the treatment for chronic pain and allergic asthma. Recently, GRC 17536, another TRPA1 antagonist, showed positive data in a Phase IIa proof of concept study in patients with painful diabetic neuropathy [47]. It is well known that many drug candidates fail in Phase IIb and Phase III. For example, the success rate in the Phase III clinical trial is only ~25–30%. The road of these drug candidates to the clinic remains long and treacherous. Finding novel uses for old drugs has been an attractive strategy for a long time. Two commercially available drugs, duloxetine and ketotifen, show potent inhibitory activity on TRPA1 channels and neuroprotective effects in OPIDN models. Since duloxetine and ketotifen are FDA-approved drugs, they could meet the urgent needs of clinical practice. In conclusion, the current study provides compelling evidence that TRPA1 mediates OPIDN and that TRPA1 is an effective therapeutic target for the neuropathy.

## Materials and methods

### Reagents

Malathion (S-[1,2-bis (ethoxycarbonyl) ethyl] O, O-dimethyl phosphorodithioate) was purchased from Sigma-Aldrich (Shanghai, China) and other OP compounds were purchased from J&K Chemicals (Shanghai, China). The AITC and HC030031 were synthesized by Shanghai Hufa chemical industry Co., Ltd (Shanghai, China) and Shanghai Biobond Pharmaceutical Co., Ltd (Shanghai, China), respectively. Duloxetine was synthesized by Shanghai Leado Pharmatech Co. Ltd, Shanghai, China. Ketotifen was synthesized by Selleck (Shanghai, China). Drug stocks were made in dimethyl sulfoxide and stored at −20 °C until use. The calcium fluorescent dye Fura-2/AM and Fluo-4/AM were purchased from Life technologies, Carlsbad, CA, USA. Collagenase from Clostridium histolyticum, Type 1A and trypsin was purchased from Sigma-Aldrich (Shanghai, China).

### Animals

All animal procedures were performed in accordance with the National Institutes of Health Guide for the Care and Use of Laboratory Animals, under protocols approved by and strictly follow guidelines of the IACUC (Institutional Animal Care and Use Committees). The IACUC checked all protocols and approved this study. Knockout mice lacking Trpa1 (C57 BL/6 background) were obtained from Dr David Julius laboratory, and the corresponding WT control mice were littermates of the *Trpa1*^−/−^. All of the knockout mice were viable and showed no developmental defects. Electrophysiological recordings and calcium imaging of DRG neurons were performed on young mice (4–5 weeks). For behavioral and pathological studies, we used adult males (10 weeks) of both the WT and knockout mice. In addition, the adult Hyline brown hens (8–12 months, 1.5–2.0 kg) that were used for behavioral and pathological studies were purchased from Shanghai XiaoWei Biological Technology Co., Ltd.

### Cell culture and transfection

Human embryonic kidney 293 (HEK293) cells were cultured in DMEM containing 10% (vol/vol) heat-inactivated fetal bovine serum, 100 units ml^−1^ penicillin–streptomycin and 2 mM l-glutamine at 37 °C in a humidity-controlled sterile incubator with 5% CO_2_. All cell culture reagents were purchased from Life Technologies. HEK293 cells were transfected transiently with the corresponding plasmids using lipofectamine 2000 (Life Technologies). Transfection efficiency was monitored by co-transfecting EGFP plasmid which codes for the enhanced green fluorescent protein. Electrophysiological recordings were performed between 24 and 36 h after transfection.

### Isolation and primary cultures of DRG neurons

DRG neurons were isolated from young C57 BL/6 mice (4–5 weeks). The sections of thoracic and lumbar DRG neurons were rapidly isolated in calcium and magnesium-free PBS with low temperature conditions (ice-bath environment). The isolated tissues were cut into pieces with scissor and incubated for 30 min with 0.1% collagenase and 0.025% trypsin which were diluted by DMEM-F12 at 37 °C. After digestion, neurons were gently dissociated using Pasteur pipettes and then plated onto poly-D-lysine pre-coated glass cover slips. DRG neurons were cultured for 24 h before use.

### Patch-clamp electrophysiology

Whole-cell patch-clamp recordings in DRG neurons (small diameter, <25 mm) and HEK293 cells were operated at room temperature (23–25 °C) using EPC-10 amplifier and Patch Master Software (HEKA). Patch pipettes had a resistance of 2–5 MΩ made of borosilicate glass capillaries and the series resistance was compensated at 60% during recording. Currents were sampled at 10 kHz and low-pass filtered at 2.9 kHz. The internal solution was as follows (in mM): 140 CsCl, 10 HEPES, 5 EGTA, 0.1 CaCl_2_ and 1 MgCl_2_, with pH adjusted to 7.2 by CsOH and osmolarity 295–300 mOsm. The calcium-free external solution contained (in mM) 140 NaCl, 5 KCl, 0.5 EGTA, 1 MgCl_2_, 10 Glucose and 10 HEPES, with pH adjusted to 7.4 by NaOH and osmolarity 300–310 mOsm. The 0.5 mM EGTA was replaced by 2 mM CaCl_2_ for the recordings in DRG neurons. TRPA1 currents development was monitored with repetitive injections of 300 ms duration voltage ramps from −100 to +100 mV every 2 s and the holding potential was set to 0 mV for 50 ms, existing in both sides of the voltage ramp. To record inward currents in DRG neurons, we conducted voltage clamp at holding membrane potential of −70 mV. Solutions were switched using a gravity-fed continuous focal perfusion system.

### Single-cell calcium imaging

Calcium influx signals were monitored using the ratio metric fluorescent indicator dye Fura-2/AM at room temperature (23–25 °C). Cells were incubated with 2 μM Fura-2/AM, which was generated by diluting the stock solution with normal external solution, for 30 min at 37 °C. Thereafter, Fura-2/AM that did not enter the cells was cleared away using external solution. The maximum excitation wave length of Fura-2/AM was changed to 340 nm from 380 nm when combined with calcium ions. Fura-2/AM was excited at 340 and 380 nm with a rapid switching monochromator obtained from TILL Photonics, and the mean fluorescence intensity ratios (F_340_/F_380_) reflected real-time changes in the concentration of intracellular free calcium ions. The external solution used in the DRG whole-cell patch-clamp recordings was also used for calcium imaging. Solutions were switched using a gravity-fed continuous focal perfusion system.

### FDSS Ca^2+^-influx assays

Ca^2+^-influx assays were carried out using HEK293 cells stably expressing mTRPA1 channels with an FDSS screening system (Hamamatsu, Japan). Before the start of the assay, HEK293-mTRPA1 cells were plated onto poly-D-Lysine (40 μg ml^−1^) coated 96-well black clear bottom plate with the cell density of 300 000 cells per milliliter in DMEM supplemented with 10% (vol/vol) fetal bovine serum, 200 μg ml^−1^ Hygromycin B, 15 μg ml^−1^ Blasticidin S HCL and 0.3 μg ml^−1^ doxycycline, which could induce the expression of mTRPA1. The 96-well plate was then put in sterile incubator overnight. The following day, Ca^2+^ influx dye Fluo-4/AM stock was diluted to 2 μM with bath solution, and added (50 μl per well) in place of the culture medium. The plate was then incubated to load the Fluo-4/AM into cells for 60 min at 37 °C in dark environment. During the incubation, we prepared the compound plate (96-well) and the margin of plate was set as blank control due to the fringing effect. At the end of dye loading period, the remnant dye was removed using bath solution to wash the 96-well cell plate twice and then add 80 μl bath solution per well.

### Behavioral testing of mice

The chosen mice were allowed to acclimate to the experimental environment for at least 2 h before testing. Intraplantar injection of 5% AITC and 30% malathion (10 μl) was achieved using separate injectors. Spontaneous pain evoked by the compounds was assessed by measuring the time (s) the mice spent on licking. For the HC030031 groups, 200 mg kg^−1^ HC030031 was intragastrically administered, and then the test was conducted after 1 h. The testing phase was divided into two parts: 0–10 min and 11–60 min.

### Assessment of delayed neuropathy in hens

The chosen adult Hyline Brown hens were raised individually in stainless steel wire cages. After 7 days of acclimatization, the chosen hens were randomly divided into groups (*n*=6–10 for each group). The hens in the delayed neuropathy groups were administered a single dose of 750 mg kg^−1^ TOCP and repeated doses of 75 mg kg^−1^ malathion by gavage. TOCP and malathion were dissolved in corn oil and administered at 0.65 ml kg^−1^. In the HC030031 treatment groups, 100 mg kg^−1^ HC030031 was administered by gavage daily beginning at day 0 or day 11. All hens were examined daily for signs of delayed neurotoxicity. A five-point graded scale was used to describe the clinical symptoms of delayed neuropathy, with 0 indicating normal ambulation; (1) suspected ataxia (lack of strength in the legs and tottering); (2) mild ataxia (slow, clumsy and unsteady gait); (3) gross ataxia (waddling gait and staggering and falling despite being active); (4) mild paralysis (typical paralytic posture and lame appearance); and (5) complete paralysis (unable to maintain posture, stand up or move at all), as described previously [44, 71, 72].

### Transmission electron microscopy imaging

For mice, subcutaneous tissue from the site of exposure was removed and fixed by immediate immersion in ice-cold 2.5% glutaraldehyde. Subsequently, the samples were post-fixed in 1% OsO_4_ for 1 h, dehydrated with an ethanol series and embedded in epon resin. Sixty-nanometer ultra-thin sections were double stained with lead citrate and uranyl acetate and examined using an electron microscopy.

For hens, the animals were anesthetized with sodium pentobarbital (100 mg kg^−1^) and killed 21 days after exposure to TOCP and malathion. They were then perfused with normal saline followed immediately by 4% paraformaldehyde. The lumbar spinal cord at approximately the L2-L3 level and the sciatic nerve of the left leg were removed and fixed by immersion in ice-cold 2.5% glutaraldehyde. Subsequently, the samples were post-fixed in 1% OsO_4_ for 1 h, dehydrated with the ethanol series, and embedded in epon resin. Sixty-nanometer ultra-thin sections were double stained with lead citrate and uranyl acetate and examined with an electron microscopy.

### High-throughput screen for TRPA1 inhibitors

The potential inhibitory activity of 2 000 commercially available drugs on mTRPA1 channels was evaluated using an IonWorks Barracuda (IWB) automated electrophysiology assay. mTRPA1-HEK293 cells were cultured in T175 flasks to ~80–90% confluence. Cells were dissociated by trypsin-EDTA for 3–5 min and then resuspended in 5 ml calcium-free external solution. The internal solution employed was the same as that used for the manual patch-clamp electrophysiology. The perforation agent amphotericin B was added to the internal solution at a concentration of 0.1 mg ml^−1^. The cells were allowed to recover for 10–15 min at room temperature before loaded into the IWB. IWB population patch-clamp 384-well plate was primed with intracellular and extracellular buffer, and 6 μl of cells (~3 million ml^−1^) was added to each well by the instrument. Electrical access was established through a 5-min incubation with amphotericin B. The sampling rate throughout the voltage protocol was 5 kHz, cells were voltage clamped at 0 mV and a 100 ms ramp protocol from −100 mV to +100 mV were used to monitor mTRPA1 currents induced by 100 μM AITC. This protocol was applied every 20 s. Data acquisition and reduction were performed using the IWB software (version 2.5.3; Molecular Devices Corporation, Union City, CA, USA). Data were corrected for leak current, and current amplitude was measured from the peak current amplitude at +100 mV. Only wells with a seal resistance >30 MΩ were included in the analysis. For pharmacological studies, each experiment included concentration-response data for HC030031 as a positive control.

### Data analysis and statistics

The data are reported as the mean±s.e.m. All statistical analyses were performed using Student’s *t*-test or two-way ANOVA (GraphPad Prism 5 Software, San Diego, CA, USA). Asterisks (*) indicate statistically significant differences from the control group (*P<0.05, **P<0.01 and ***P<0.001).

## Figures and Tables

**Table 1:**
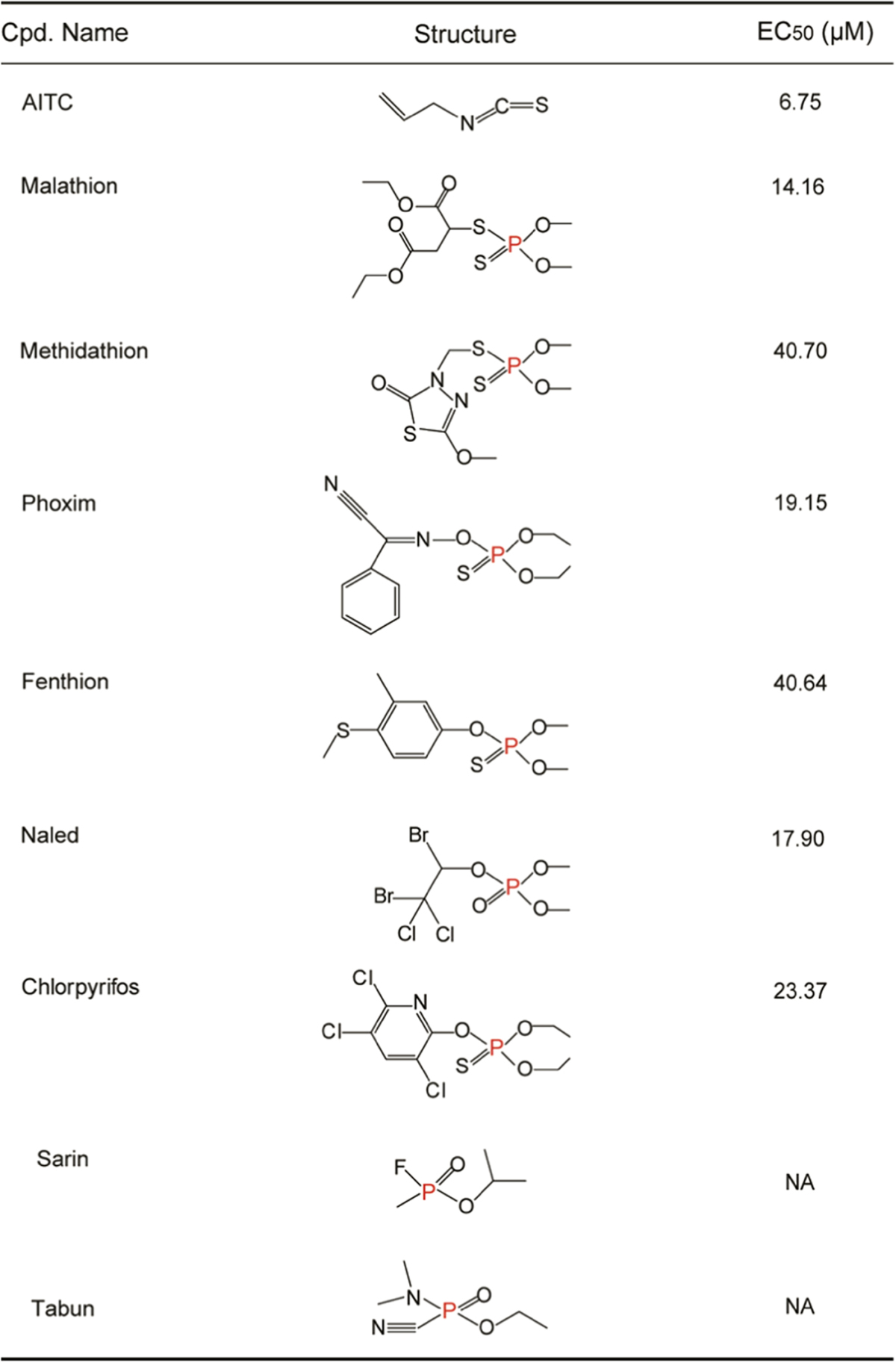
The structure and activation potency (EC_50_, μM) of AITC and OPs on mTRPA1

mTRPA1 channels were stably expressed in HEK293 cells and the EC_50_ values were determined by measuring change in [Ca^2+^]_i_. NA indicated the EC_50_ was not tested.
