# Supplementary material for: TRPA1 channel mediates organophosphate-induced delayed neuropathy
Source: Cell Discov. 2017 Aug 1;3:17024–. doi: 10.1038/celldisc.2017.24 (PMC5537602; doi:10.1038/celldisc.2017.24)
Supplement: Supplementary Figures [file celldisc201724-s1.pdf]

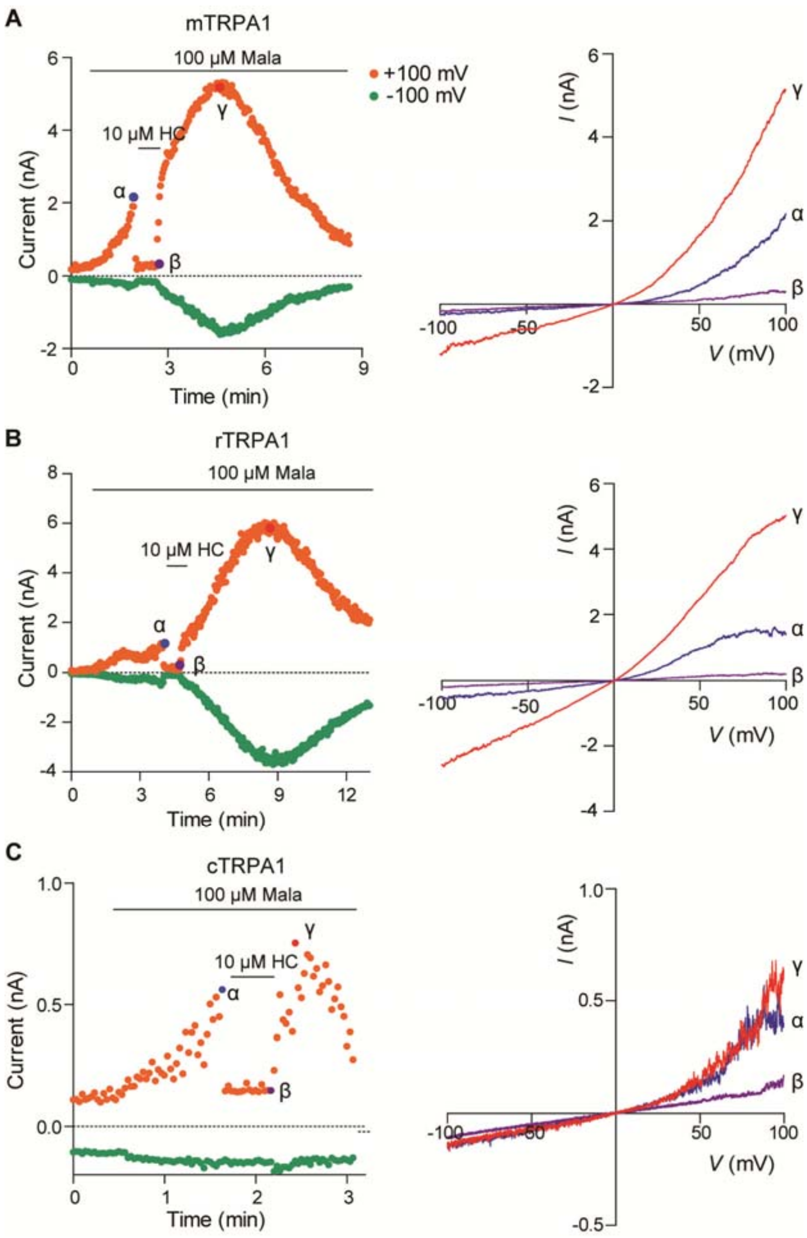

Figure S1. Malathion-induced whole-cell current of mouse (m), rat (r) and chicken (c) TRPA1 channels expressed in HEK293 cells. (**A-C**) Malathion (Mala) activates mTRPA1 (**A**), rTRPA1 (**B**) and cTRPA1(**C**) channels in calcium-free external solution. The TRPA1 currents were blocked by 10 µM HC030031 (HC). The currents measured at -100 mV (green circles) and +100 mV (orange circles) during each ramp is plotted as a function of time and the current-voltage (I-V) relationships before (α, blue) and after (β, purple) treatment with HC030031 and the peak of malathion response (γ, red). Similar activation currents were

observed in more than 5 cells for each species TRPA1 channels.

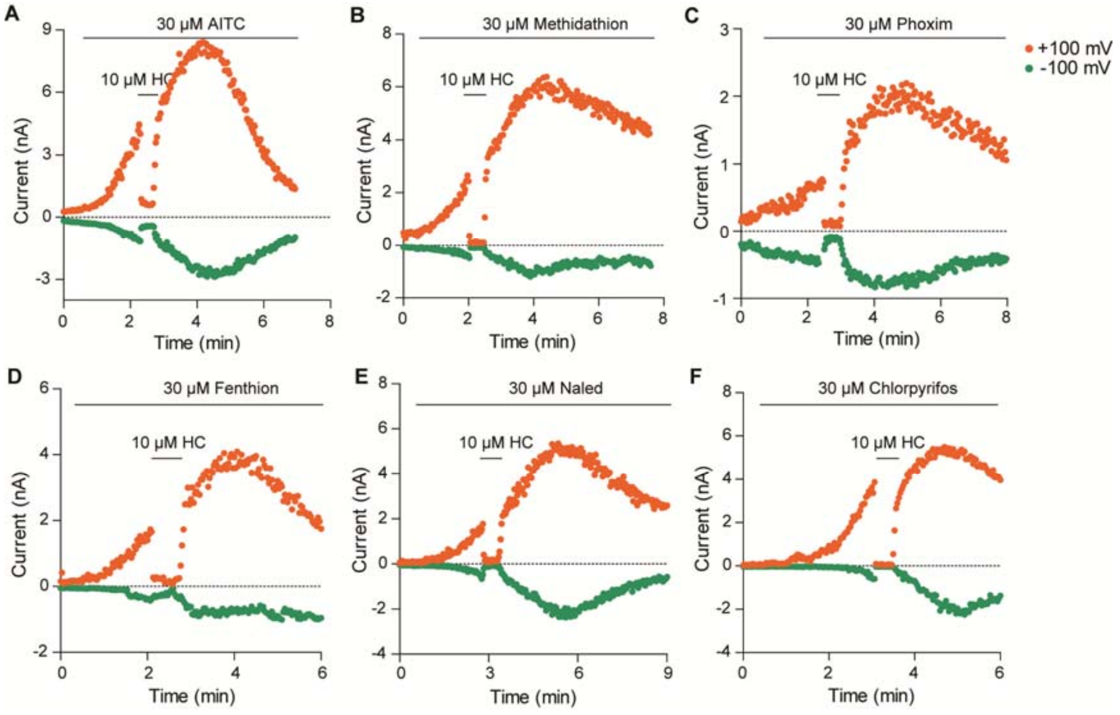

Figure S2. Activation of hTRPA1 induced by AITC and OPs. (**A-F**) Whole-cell current recording of hTRPA1 channels activated by AITC (**A**) or the OP compounds methidathion (**B**), phoxim (**C**), fenthion (**D**), naled (**E**) and chlorpyrifos (**F**) in calcium-free external solution. The TRPA1 currents were blocked by 10 μM HC030031 (HC). The currents measured at -100 mV (green circles) and +100 mV (orange circles) during each ramp are plotted as a function of time. Similar activation currents were observed in more than 4 cells for AITC and each tested OP.

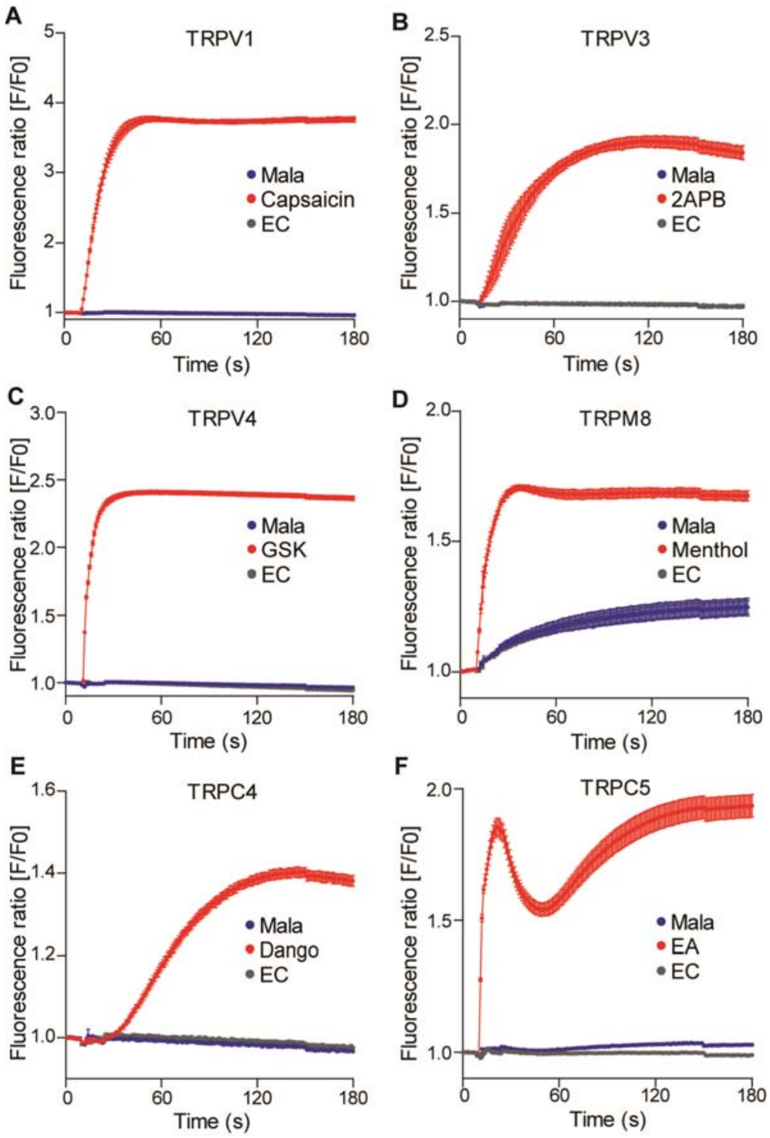

Figure S3. Specificity of malathion for TRPA1 channels. (**A**-**F**) Time course of the fluorescence signal induced by malathion (Mala, 10 μM) and the indicated positive agonists (10 μM) in HEK293 cells overexpressing TRPV1 (**A**), TRPV3 (**B**), TRPV4 (**C**), TRPM8 (**D**), TRPC4 (**E**) and TRPC5 (**F**) channels. The fluorescence signals are scaled as F/F_0. EC indicates external solution. Similar fluorescence signal results were observed in more than 3 cells for each tested TRP channel. All bars are presented as the mean ± SEM.

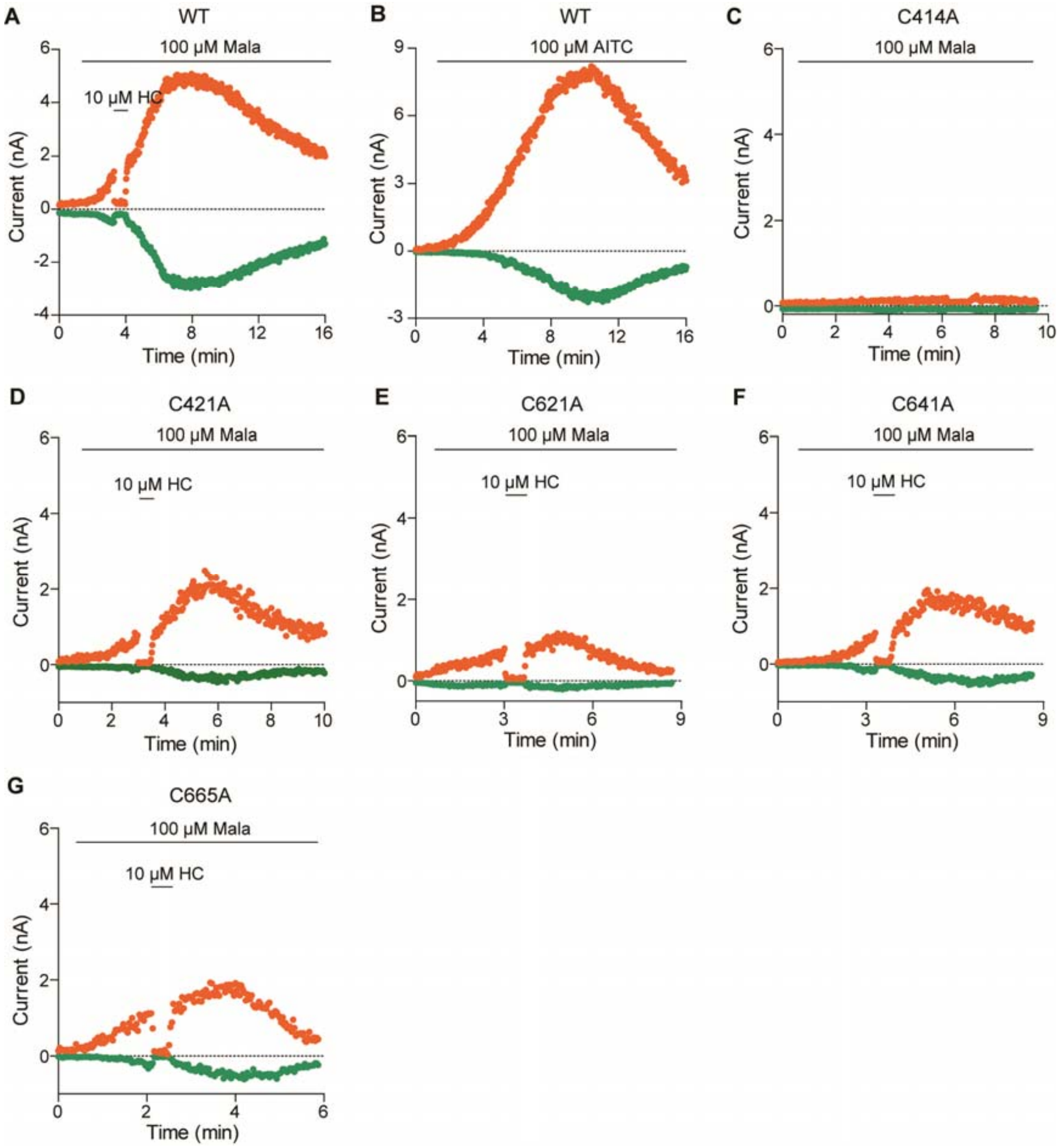

Figure S4. The molecular basis of malathion-mediated TRPA1 activation. (**A**, **B**) I-T plots of WT hTRPA1 activation by 100 µM malathion (**A**) or 100 µM AITC (**B**). (**C-G**) I-T plots of the activation of C414A (**C**), C421A (**D**), C621A (**E**), C641A (**F**) and C665A (**G**) mutants by 100 µM malathion. The WT and mutant hTRPA1 currents were blocked by 10 µM HC030031 (HC). Similar activation currents were observed in more than 5 cells for each construct.

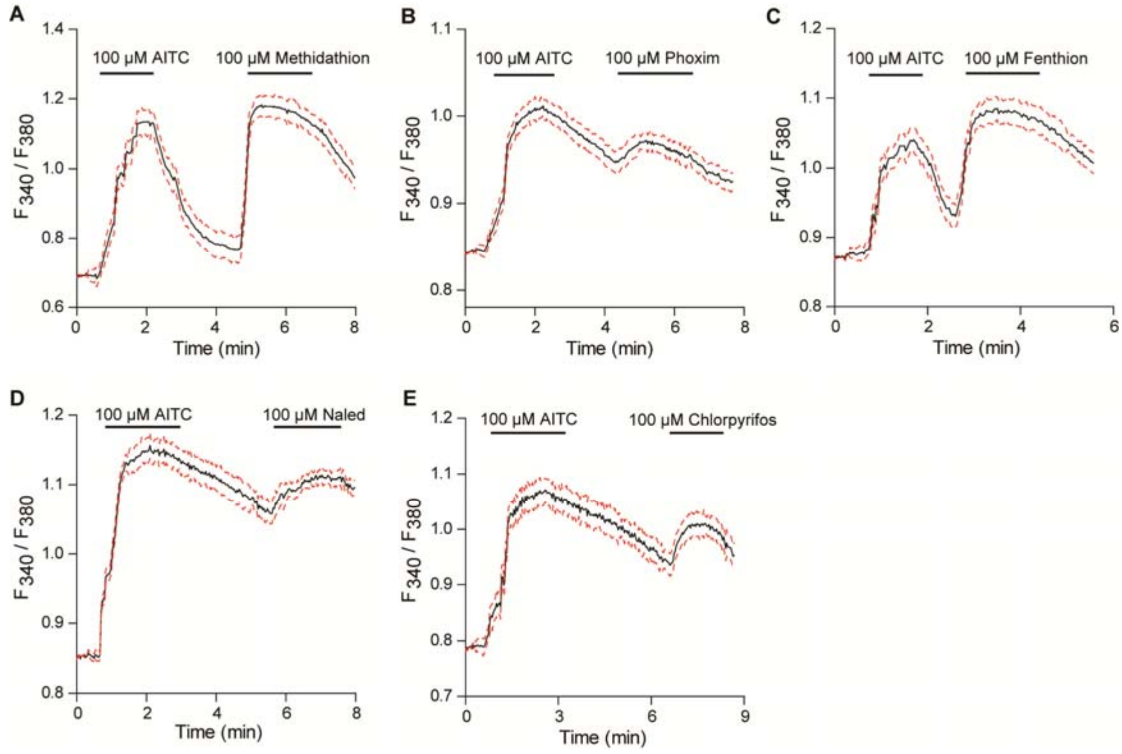

Figure S5. OP-induced fluorescence signals in DRG neurons. Changes in the Fura-2/AM ratio in DRG neurons from WT mice after the application of AITC or methidathion (**A**), phoxim (**B**), fenthion (**C**), naled (**D**) or chlorpyrifos (**E**). Similar changes were observed in more than 190 neurons for each tested OP. All bars are presented as the mean ± SEM.

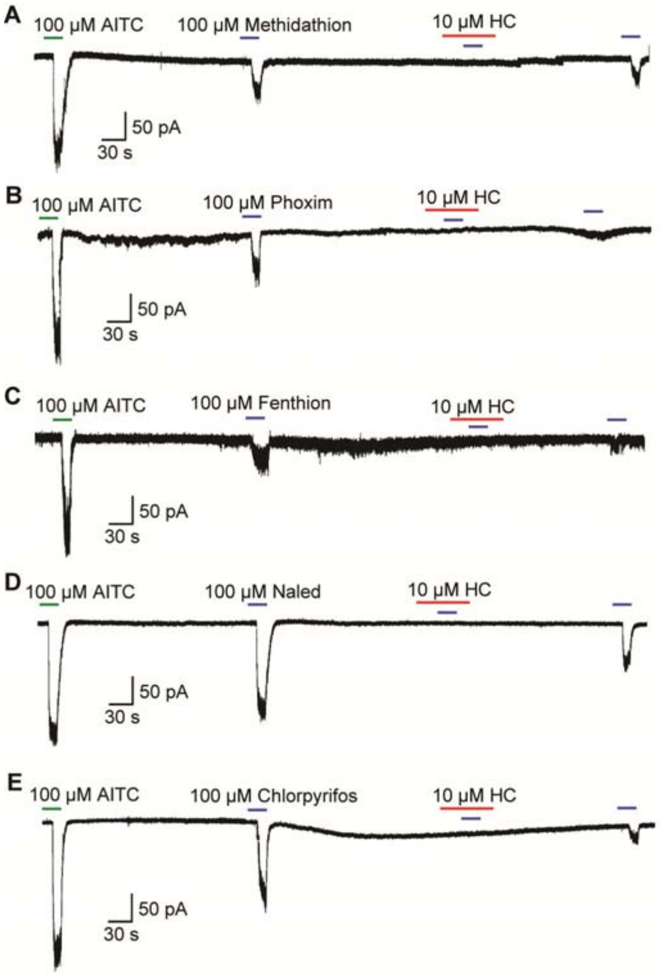

Figure S6. OP-induced inward currents in DRG neurons. AITC and OPs (methidathion (**A**), phoxim (**B**), fenthion (**C**), naled (**D**) or chlorpyrifos (**E**)) induced inward currents in small-sized DRG neurons via TRPA1 channels. The elicited currents were blocked by 10 μM HC030031. The green, blue and red bars showed the AITC, indicated OPs and HC030031 (HC) application periods, respectively.

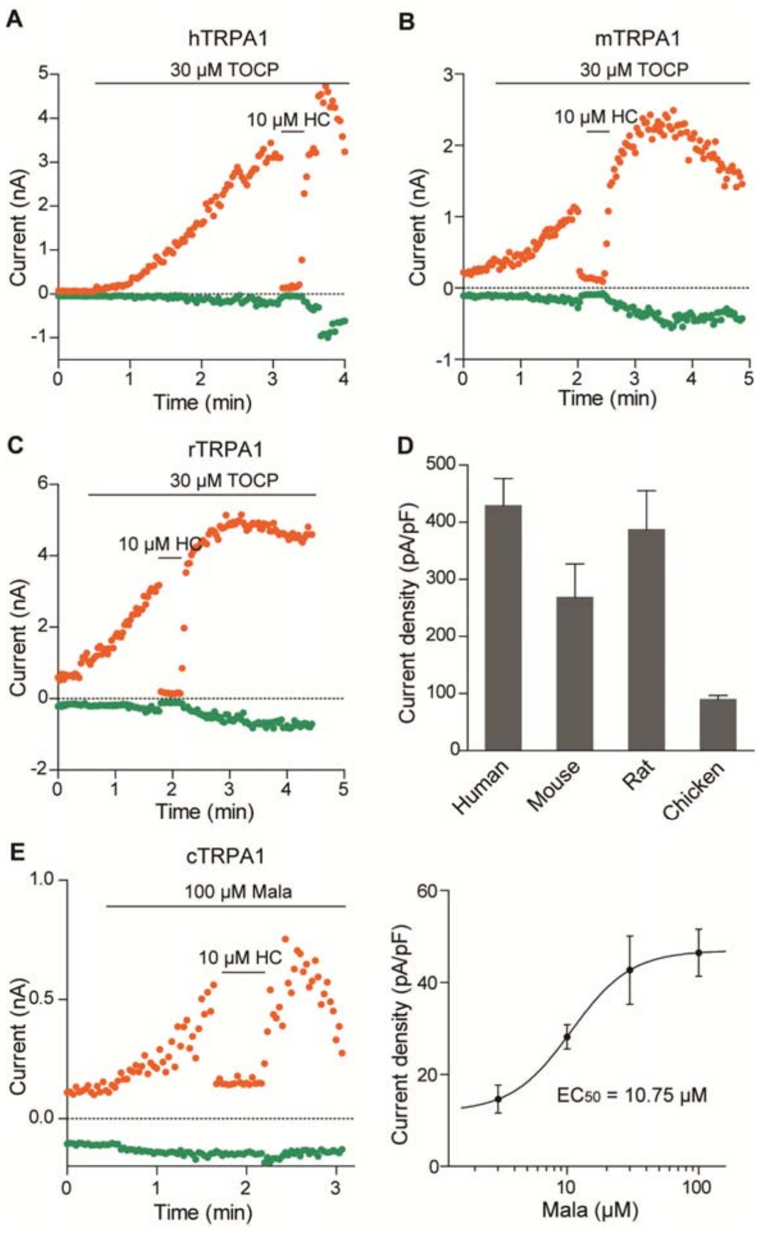

Figure S7. TOCP activates mouse, rat and human TRPA1 channels. (**A**-**C**) Whole-cell recording of the activation of hTRPA1 (**A**), mTRPA1 (**B**) and rTRPA1 (**C**) channels by 30 μM TOCP in calcium-free external solution. The TRPA1 currents were blocked by 10 μM HC030031 (HC). The currents measured at -100 mV (green circles) and +100 mV (orange circles) during each ramp are plotted as a function of time. (**D**) Summary of current density of human, mouse, rat and chicken TRPA1 channels induced by 30 μM TOCP. (**E**) Malathion dose-dependently activates cTRPA1. The TRPA1 currents were blocked by 10 μM

HC030031 (HC). Similar activation currents were observed in more than 4 cells for each test.

All bars are presented as the mean ± SEM.

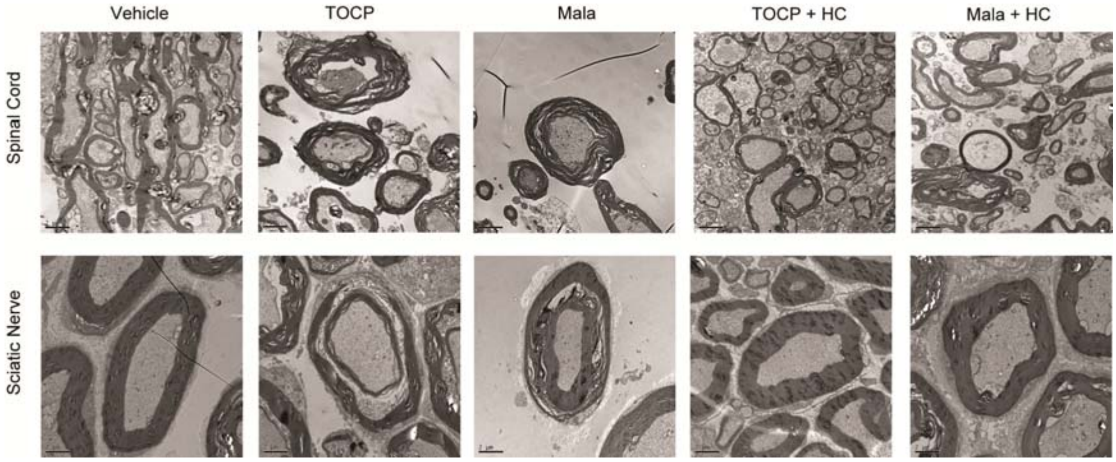

Figure S8. TEM images of hen spinal cords (upper row) and sciatic nerves (lower row) from the various groups (5000x). The neuropathological changes in spinal cord and sciatic nerve for TOCP, malathion (Mala), TOCP + HC and Mala + HC groups compared with vehicle group. Both TOCP and malathion severely damaged the spinal cord and sciatic nerve, inducing large-scale avulsion and dissolution of the myelin sheaths, causing atrophy or content loss of the nerve fibers and leading to the accumulation of double-membrane autophagosomes in axons. Due to the abnormal accumulation of autophagic vacuoles and autophagosomes, the damaged axons displayed swellings. And the nerve injuries were significantly reduced in HC030031-treated groups.

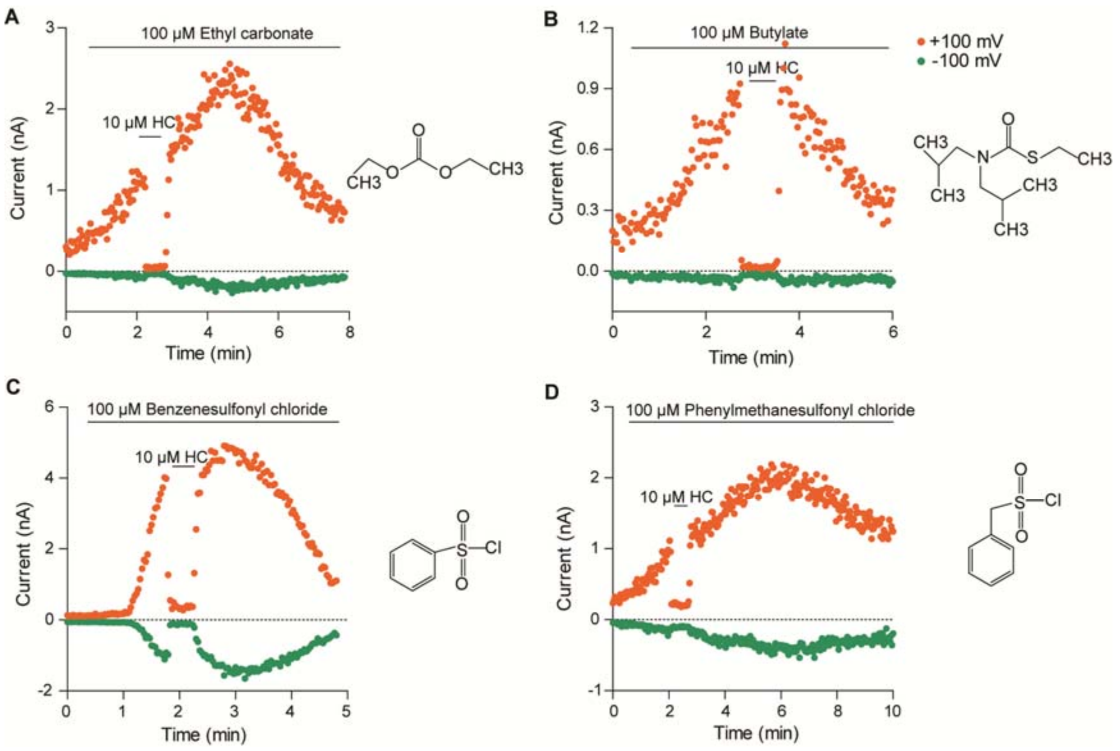

Figure S9. NTE inhibitors activate hTRPA1 channels. (**A-D**) Whole-cell current recording of hTRPA1 channels activated by 100 μM Ethyl carbonate (**A**), Butylate (**B**), Benzenesulfonyl chloride (**C**) and Phenylmethanesulfonyl chloride (**D**). The elicited currents could be blocked by 10 μM HC030031. The structure of the esterase inhibitors are displayed concomitantly. Similar activation currents were observed in more than 4 cells for each test.

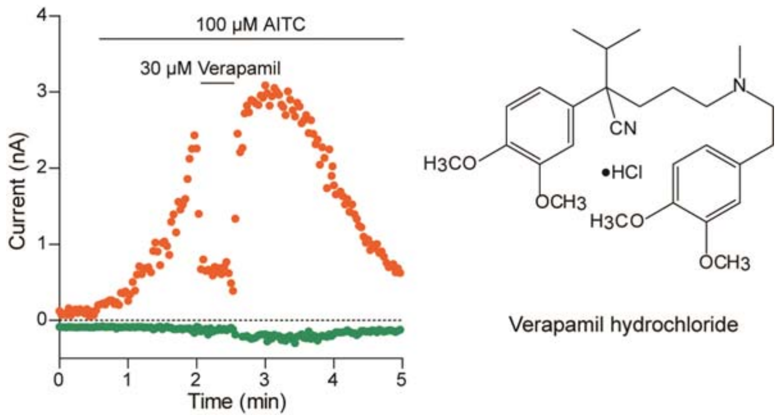

Figure S10. Voltage-gated calcium channel blocker verapamil inhibits hTRPA1 channel. The hTRPA1 currents activated by 100 μM AITC could be blocked by 30 μM verapamil. The structure of verapamil is displayed concomitantly. Similar inhibitory effects were observed in more than 4 cells.
